# Deep Learning Methodology for Differentiating Glioma Recurrence From Radiation Necrosis Using Multimodal Magnetic Resonance Imaging: Algorithm Development and Validation

**DOI:** 10.2196/19805

**Published:** 2020-11-17

**Authors:** Yang Gao, Xiong Xiao, Bangcheng Han, Guilin Li, Xiaolin Ning, Defeng Wang, Weidong Cai, Ron Kikinis, Shlomo Berkovsky, Antonio Di Ieva, Liwei Zhang, Nan Ji, Sidong Liu

**Affiliations:** 1 Beijing Academy of Quantum Information Sciences Beijing China; 2 Department of Neurosurgery Beijing Tiantan Hospital Capital Medical University Beijing China; 3 School of Instrumentation and Optoelectronic Engineering Beihang University Beijing China; 4 Department of Neuropathology Beijing Neurosurgical Institute Capital Medical University Beijing China; 5 School of Computer Science The University of Sydney Sydney Australia; 6 Surgical Planning Laboratory, Department of Radiology Brigham and Women’s Hospital Harvard Medical School Boston, MA United States; 7 Department of Computer Science University of Bremen Bremen Germany; 8 Fraunhofer Institute for Digital Medicine MEVIS Bremen Germany; 9 Centre for Health Informatics, Australian Institute of Health Innovation Macquarie University Sydney Australia; 10 Computational NeuroSurgery Lab Department of Clinical Medicine, Faculty of Medicine and Health Sciences Macquarie University Sydney Australia

**Keywords:** recurrent tumor, radiation necrosis, progression, pseudoprogression, multimodal MRI, deep learning

## Abstract

**Background:**

The radiological differential diagnosis between tumor recurrence and radiation-induced necrosis (ie, pseudoprogression) is of paramount importance in the management of glioma patients.

**Objective:**

This research aims to develop a deep learning methodology for automated differentiation of tumor recurrence from radiation necrosis based on routine magnetic resonance imaging (MRI) scans.

**Methods:**

In this retrospective study, 146 patients who underwent radiation therapy after glioma resection and presented with suspected recurrent lesions at the follow-up MRI examination were selected for analysis. Routine MRI scans were acquired from each patient, including T1, T2, and gadolinium-contrast-enhanced T1 sequences. Of those cases, 96 (65.8%) were confirmed as glioma recurrence on postsurgical pathological examination, while 50 (34.2%) were diagnosed as necrosis. A light-weighted deep neural network (DNN) (ie, efficient radionecrosis neural network [ERN-Net]) was proposed to learn radiological features of gliomas and necrosis from MRI scans. Sensitivity, specificity, accuracy, and area under the curve (AUC) were used to evaluate performance of the model in both image-wise and subject-wise classifications. Preoperative diagnostic performance of the model was also compared to that of the state-of-the-art DNN models and five experienced neurosurgeons.

**Results:**

DNN models based on multimodal MRI outperformed single-modal models. ERN-Net achieved the highest AUC in both image-wise (0.915) and subject-wise (0.958) classification tasks. The evaluated DNN models achieved an average sensitivity of 0.947 (SD 0.033), specificity of 0.817 (SD 0.075), and accuracy of 0.903 (SD 0.026), which were significantly better than the tested neurosurgeons (*P*=.02 in sensitivity and *P*<.001 in specificity and accuracy).

**Conclusions:**

Deep learning offers a useful computational tool for the differential diagnosis between recurrent gliomas and necrosis. The proposed ERN-Net model, a simple and effective DNN model, achieved excellent performance on routine MRI scans and showed a high clinical applicability.

## Introduction

Brain radiation necrosis (ie, pseudoprogression) can be a consequence of radiation therapy, which is used for the treatment of brain tumors, with an incidence of 3%-24% [1-4]. It is of paramount importance to distinguish radiation necrosis from tumor recurrence, as these two pathologies share similar appearances in neuroimaging yet have different treatments and outcomes [5,6]. Currently, various imaging modalities, such as magnetic resonance spectroscopy (MRS) [7,8], perfusion-weighted imaging (PWI) [9], diffusion-weighted imaging (DWI) [10], and positron emission tomography (PET) with different tracers [11,12], have been applied for differentiating radiation necrosis from tumor recurrence; yet their efficacy and reliability still need further validation. Differential diagnosis between recurrent tumors and necrosis remains a major challenge in neuro-oncology and neuroradiology [1,2,5,6,13].

**Figure 1:**
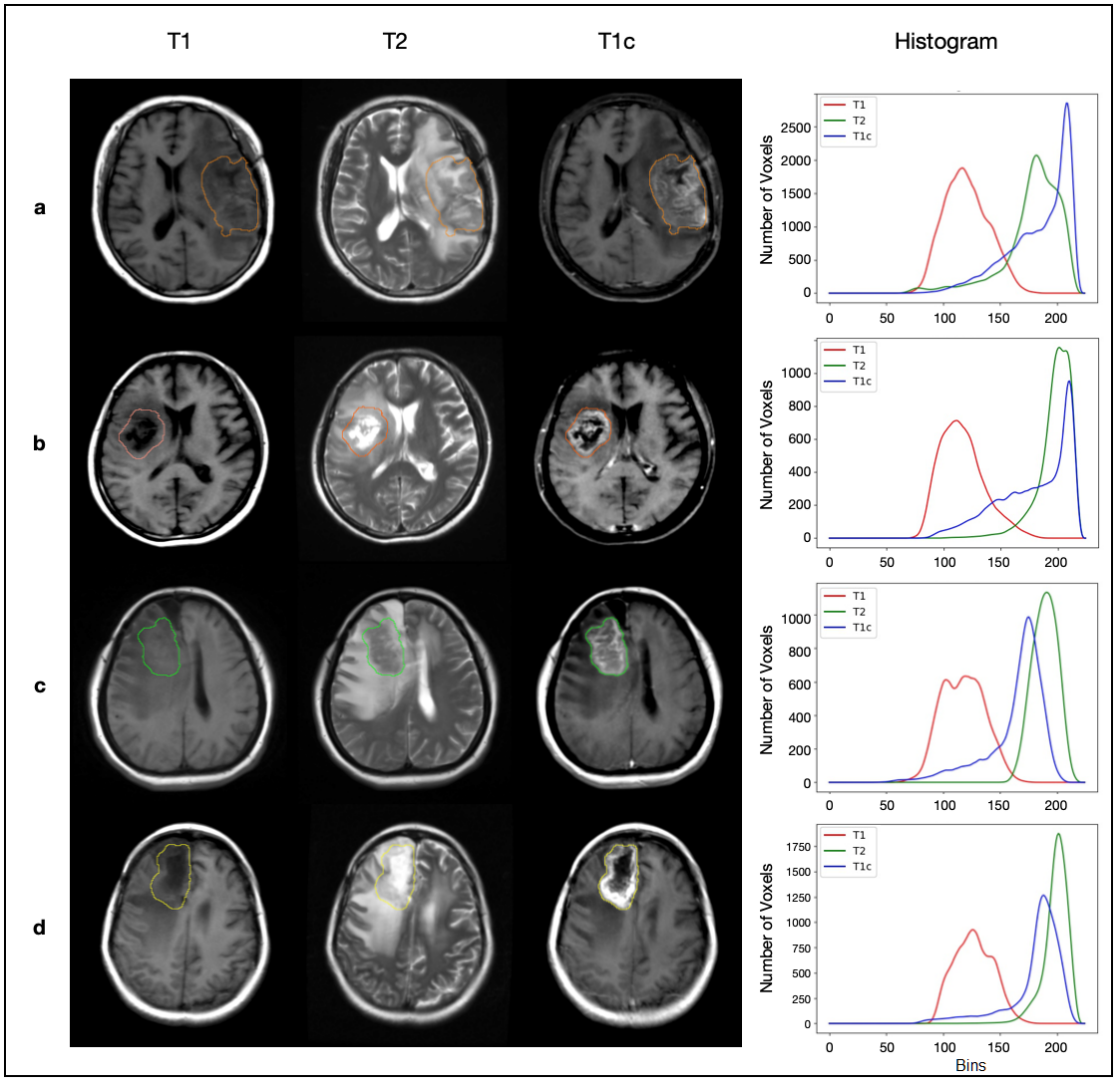
The T1, T2, and T1c magnetic resonance imaging (MRI) sequences of 4 patients with their histograms of the voxels within the lesion masks. Patients (a) and (b) represent recurrent tumors; patients (c) and (d) represent radionecrosis lesions. The lesion masks were manually drawn using the software ITK-SNAP, generally used for delineating regions of interest. The histograms were created for individual sequences and further smoothed using the Hann filter. ITK: Insight Toolkit. T1: T1-weighted MRI; T1c: gadolinium-contrast-enhanced T1-weighted MRI; T2: T2-weighted MRI.

Recent studies demonstrate that although radiologists may not be able to systematically identify differences in the highly variable appearances of brain tumors and radionecrosis, handcrafted features extracted from routine magnetic resonance imaging (MRI) can effectively differentiate these two conditions [14-16]. As shown in these studies, handcrafted radiomic features can capture the variations in image intensity, shape, and volume and have shown promising results (see Figure 1). However, there are two major limitations that may restrict the use of these methods in the clinical setting. The first limitation is that all these methods require manual segmentation of the lesion (ie, drawing regions of interest [ROIs] of the lesion on T1-weighted MRI [T1], gadolinium-contrast-enhanced T1 [T1c], and/or T2-weighted MRI [T2]/fluid-attenuated inversion recovery [FLAIR]), from which the texture or shape features can be extracted [17]. The ROI segmentation is time-consuming and operator dependent, introducing human interference and potential noise into the analysis. Furthermore, handcrafted features extracted in these studies are usually redundant and require feature selection, which, if inaccurate, may bias the analysis.

Deep learning is a data-driven approach that uses deep neural network (DNN) models to learn the feature representations at multiple levels of abstraction [18]. Deep learning models, such as Visual Geometry Group (VGG) [19], residual neural network (ResNet) [20], and Inception [21], have substantially improved the state of the art in many visual analysis tasks (eg, ImageNet Large Scale Visual Recognition Challenge [22]), compared to handcrafted features. Deep learning methods have also demonstrated human-level performance in medical image computing, such as skin cancer classification [23], diabetic retinopathy grading [24], glaucoma detection [25], early diagnosis of Alzheimer disease [26], and, most recently, COVID-19 severity assessment [27]. Yet to the best of our knowledge, the application of deep learning in differentiating glioma recurrence from postradiotherapy necrosis has not been investigated so far.

Therefore, in this work we aim to explore the potential benefit of deep learning algorithms for distinguishing between radionecrosis and tumor recurrence using routine MRI scans. We proposed a novel DNN model (ie, efficient radionecrosis neural network [ERN-Net]) to automatically characterize the features of gliomas and necrosis from MRI images and to classify the lesions at image-based and subject-based levels, which outperformed the human experts (ie, neurosurgeons) and the state-of-the-art DNN models. Furthermore, the proposed method does not depend on lesion segmentation or any handcrafted features and, therefore, may have a higher clinical applicability.

## Methods

### Patient Data and Imaging Protocol

**Figure 2:**
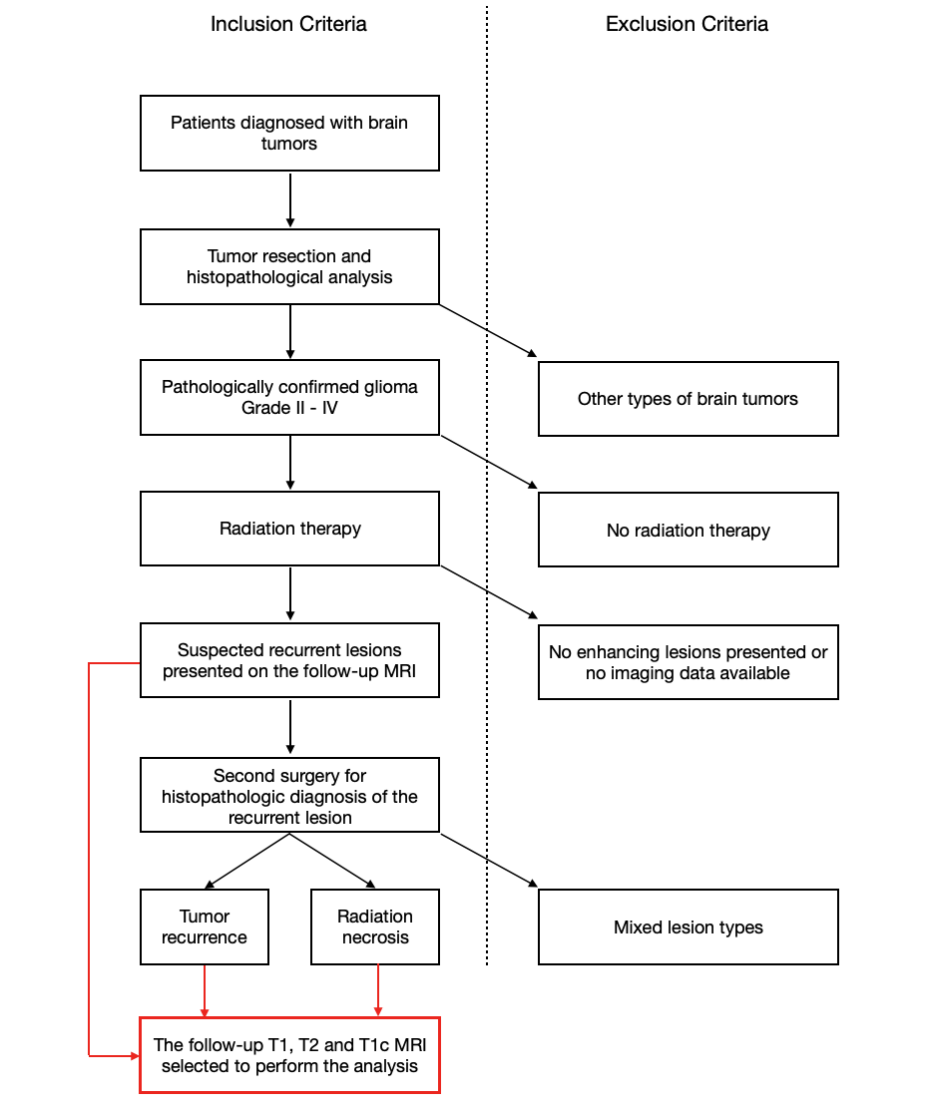
The selection process for the patient cohorts in this study. MRI: magnetic resonance imaging; T1: T1-weighted MRI; T1c: gadolinium-contrast-enhanced T1-weighted MRI; T2: T2-weighted MRI.

This study was approved by the Institutional Review Board of Beijing Tiantan Hospital, Capital Medical University (BTH-CMU), China, and the requirement for informed consent was waived by the board as this research involves no more than minimal risk. The criteria for selecting the patient cohorts are shown in Figure 2.

We retrospectively identified patients who underwent brain tumor resection between January 2010 and November 2018, confirmed by pathology examination to be gliomas. Among the selected patients, we further selected the ones who underwent subsequent radiation therapy and presented with suspected recurrent lesions on radiological follow-up. All the patients included in this study underwent a second surgery to differentiate glioma recurrence from radiation necrosis. Histopathologic diagnoses of both the initial and recurrent lesions were performed by neuropathologists at BTH-CMU. Patients were excluded from the study if their histopathological analyses showed a mixture of tumor and necrosis.

A cohort of 146 patients were identified using our criteria. Of those, 96 (65.8%) patients were diagnosed to be affected by recurrent glioma, and 50 (34.2%) by necrosis. Of the 146 patients, 117 subjects (80.1%) were randomly assigned to the training set, and the remaining 29 subjects (19.9%) were retained as the test set. It is a common practice to split the cohort into a training set and a test set in machine learning studies, and the training set to test set ratio usually varies from 60:40 to 90:10 [20,23,26]. In this study, we chose the 80:20 split ratio to balance the number of cases that can be used to train the model and the workload on the human experts to assess the test cases. Table 1 shows the demographic data of the subjects in the cohort as well as the distribution of the cases in the training and test data sets. The histopathological analysis results of the recurrent lesions, either recurrent tumor or necrosis, were used to categorize patients’ imaging data.

**Table 1:** Demographic and clinical data of the patient cohorts enrolled in this study.

Characteristic		Training set (n=117)	Test set (n=29)	Total (N=146)
Sample size (N=146), n (%)		117 (80.1)	29 (19.9)	146 (100)
Age in years, mean (SD)		40.9 (12.4)	42.0 (9.9)	41.1 (11.9)
**Gender, n (%)**				
	Male	63 (53.8)	15 (52)	78 (53.4)
	Female	54 (46.2)	14 (48)	68 (46.6)
**Diagnosis of primary lesion, n (%)**				
	Grade II	33 (28.2)	8 (28)	41 (28.1)
	Grade III	26 (22.2)	6 (21)	32 (21.9)
	Grade IV	45 (38.5)	11 (38)	56 (38.4)
	Unknown	13 (11.1)	4 (14)	17 (11.6)
**Diagnosis of recurrent lesion, n (%)**				
	Necrosis	40 (34.2)	10 (34)	50 (34.2)
	Glioma	77 (65.8)	19 (66)	96 (65.8)

The follow-up MRI scans of the identified patients prior to the second surgery for histopathologic diagnosis were selected to perform the analysis. The MRI data were acquired from five MRI systems at BTH-CMU. The specifications of the imaging data are listed in Table 2. All the patients have the axial T1, T2, and T1c sequences, acquired during routine clinical visits. A total of 42 MRI scans were acquired using the MAGNETOM Trio, A Tim system (Siemens), 28 scans using the MAGNETOM Verio system (Siemens), 25 scans using the Discovery MR750 system (GE Healthcare), 29 scans using the GENESIS SIGNA system (GE Healthcare) with 3 T magnetic field, and 22 using the SIGNA system (GE Healthcare) with 1.5 T magnetic field.

**Table 2:** Specifications of the imaging data acquired from the different magnetic resonance imaging systems.

Imaging system	Field of view, mm	Slice thickness, mm	Slice spacing, mm	Matrix size
Siemens MAGNETOM Trio Tim	220	5.0	6.5	496 × 512
Siemens MAGNETOM Verio	220	5.0	6.0	496 × 512
GE Healthcare Discovery MR750	240	5.0	6.5	512 × 512
GE Healthcare GENESIS SIGNA 3 T	240	5.0	6.0	512 × 512
GE Healthcare SIGNA 1.5 T	240	5.5	6.5	512 × 512

### Data Preprocessing

To standardize the MRI data across multiple MRI systems, the following preprocessing pipeline was used. First, the imaging data were corrected for bias field using the improved nonparametric, nonuniform-intensity normalization algorithm [28] built into the Advanced Normalization Tools suite of tools for brain and image analysis [29]. Second, for every patient’s MRI data, the T1c and T2 images were coregistered to the T1 space using the Functional Magnetic Resonance Imaging of the Brain (FMRIB) Software Library (FSL) FMRIB Linear Image Registration Tool (FLIRT) pipeline with a 6-degree-of-freedom transform [30,31]. Finally, the magnetic resonance images were linearly mapped and resampled to the Montreal Neurological Institute 152 template [32], also using FSL FLIRT, in order to make the dimensions and orientation of all the images uniform.

**Figure 3:**
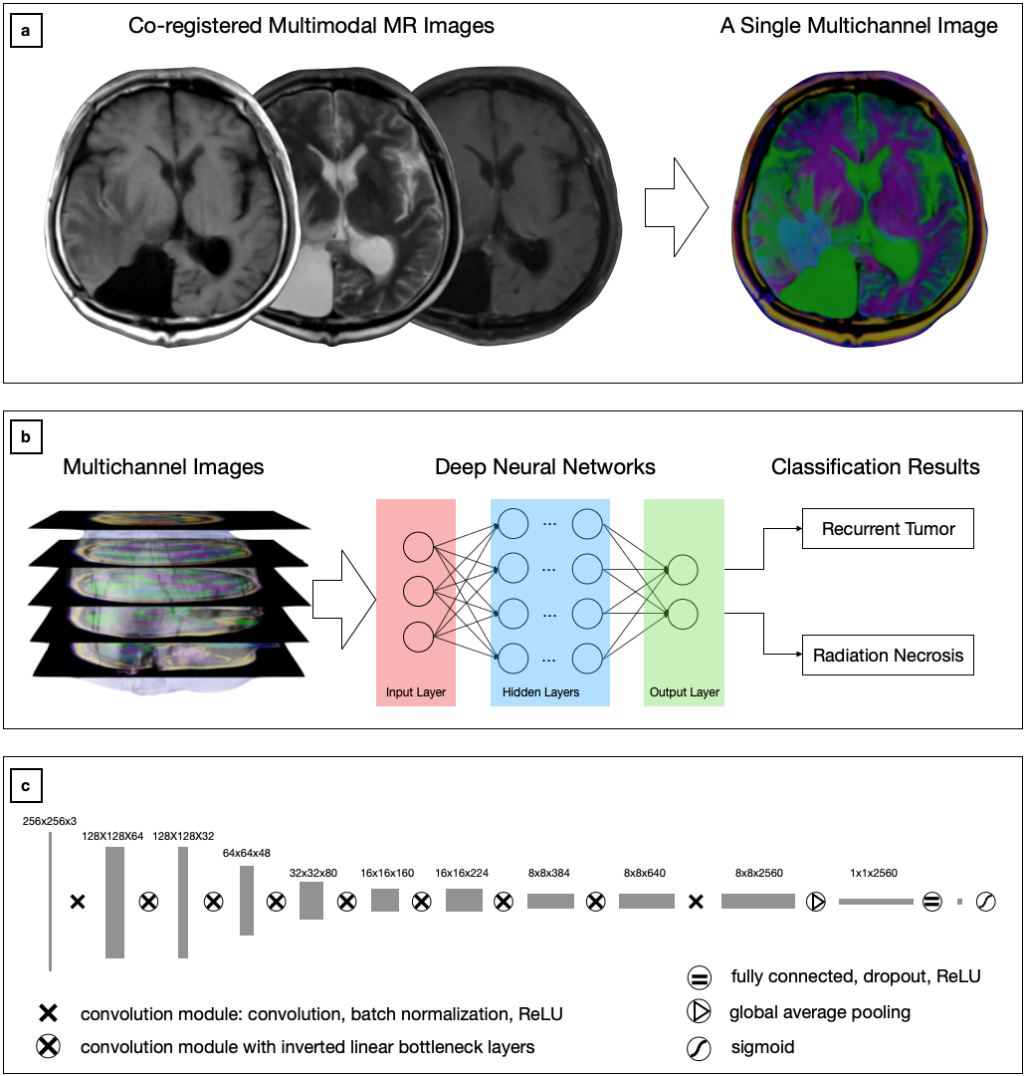
Overview of the proposed approach. (a) The co-registered multimodal images were fused as a multichannel RGB image with T1, T2, and T1c images representing the Red, Green and Blue channels, respectively. (b) The multichannel magnetic resonance (MR) images were used to train the deep neural network (DNN) models that classified the test MR images as either a recurrent tumor or radiation necrosis. (c) Architecture of the proposed efficient radionecrosis neural network (ERN-Net). ReLU: rectified linear unit; T1: T1-weighted magnetic resonance imaging (MRI); T1c: gadolinium-contrast-enhanced T1-weighted MRI; T2: T2-weighted MRI.

The MRI slices presenting enhancing lesions were identified by neuroradiologists or neurosurgeons; the multimodal magnetic resonance slices—T1, T2, and T1c—were then fused into multichannel images, as shown in Figure 3 (a). To minimize the interrater variance, we requested that the radiologists and neurosurgeons use 3D Slicer, version 4.6.2 [33], to place a marker on the axial slices if they saw a suspected recurrent lesion on the slice. Therefore, no manual outlining of the lesion was performed, taking less than two minutes for a radiologist to review an MRI image and identify the slices containing the lesion. These annotations provided by experienced neurosurgeons were used as the *ground truth* for evaluating the performance of the classifier.

The MRI slices containing the lesion were identified manually by an experienced neurosurgeon (NJ) and further reviewed by an imaging analyst (YG) based on the T1c scans. The axial T1c and corresponding T2 and T1 slices were then saved as 2D multichannel images for further analysis. A total of 5824 multichannel images, each consisting of a T1, a T2, and a T1c slice, were extracted from the 117 patients in the training set, and 1472 multichannel images were extracted from the 29 patients in the test set. The multichannel images were used to train the DNN models, which were subsequently applied to predict the patients’ lesion types in the test set based on their imaging data, as shown in Figure 3 (b).

### Efficient Radionecrosis Neural Network

DNN models can be considered as mathematical functions with numerous parameters. For image classification, DNN models usually use pixel values as the input features. The neurons in the hidden layers of the DNN are responsible for transforming lower-level features to higher-level features that can be used for classification. While training a DNN model, the training images and diagnostic labels (dichotomized; 0: radiation necrosis; and 1: tumor recurrence) are used to update the parameters of the model. At each training step, the model predicts the diagnostic label for an input training image, then the prediction is compared to its ground truth label, such that the parameters of the model are modified to reduce the error on that image prediction. This process is then repeated for every image in the training set over many iterations to let the model “learn” how to differentiate the tumor recurrence signature from the necrosis one in magnetic resonance images. After the model is fully trained, it is used to infer the diagnostic probability distribution of necrosis and tumor recurrence for the test images.

For feature learning and classification, we proposed a light-weighted DNN model (ie, ERN-Net) to learn radiological features of gliomas and necrosis from MRI scans. The proposed ERN-Net model, as illustrated in Figure 3 (c), consists of only nine convolutional modules, including seven with inverted linear bottleneck layers [34]. We also benchmarked five state-of-the-art DNN models: VGG16 and VGG19 [19], ResNet-50 [20], Inception-v3 [21], and Inception-ResNet-v2 [35]. It is noteworthy that ERN-Net is 3 times smaller and 8.1 times faster than Inception-v3 [36]. All the DNN models were implemented using the TensorFlow framework, version 1.14 [37], with the ImageNet pretrained weights imported from the Keras library [38]. To address the imbalanced sample distribution, we assigned different weights to the classes during the training phase based on the ratio between the number of samples in each class and the total number of samples scaled by the number of classes (necrosis: 1.5; recurrence: 0.75). A more detailed description of these DNN models can be found in Multimedia Appendix 1.

### Performance Evaluation

To evaluate the performance of the DNN models on image-wise classification, we designed an experiment in which we trained and tested these DNN models on the same data set, including 5824 training images and 1472 test images. This experiment was carried out on a per-image basis, with each image treated as an individual input sample. We also compared the performance of single-modal and multimodal MRI in the image-wise classification task. Sensitivity, specificity, accuracy, and area under the curve (AUC) of the receiver operating characteristic (ROC) curve were used to evaluate the classification performance.

To evaluate the performance of the DNN models on a subject basis, we designed another experiment in which we aggregated the image-wise classification results to infer each subject’s diagnosis. For each subject in the test set, the models that had been trained for image-wise classification in the previous experiment were reused to classify the stack of the subject’s images; the image-wise classification results were then averaged as the output prediction of that subject. Performances of these DNN models in subject-wise classification were also compared with those of the human experts.

## Results

### Image-wise Classification

Table 3 shows the summary of the comparison of different MRI sequences using DNN models. T1c was the best performing sequence among the three routine MRI sequences, with consistently higher accuracy and AUC than T1 and T2 sequences across all the DNN models. T1c also achieved the highest sensitivity with VGG16 and Inception-v3 models (0.874 and 0.769, respectively), and the highest specificity with VGG19 and ResNet-50 models (both equal to 0.653). Considering AUC as a single metric that combines sensitivity and specificity, T2 performed slightly better than T1, although there was disagreement in other evaluation metrics. ERN-Net outperformed the VGG models in AUC on T1c (0.807, 95% CI 0.782-0.832), while Inception-ResNet-v2 achieved the highest AUC (0.841, 95% CI 0.818-0.864). We found that the sensitivity was higher than specificity in most models and sequences. This can be partially explained by the imbalanced sample distribution in the two classes, which might bias the models and, hence, the classification results.

Table 4 shows the performance comparison of the DNN models on multimodal MRI images. ERN-NET had the highest AUC (0.915, 95% CI 0.895-0.932), which was slightly better than Inception-ResNet-v2 (0.913, 95% CI 0.895-0.931) and substantially better than the other DNN models. Inception-ResNet-v2 achieved the highest score in sensitivity (0.925, 95% CI 0.907-0.941) and accuracy (0.867, 95% CI 0.848-0.884), while VGG16 had the highest specificity (0.826, 95% CI 0.791-0.858). The DNN models based on multimodal MRI outperformed the models based on individual MRI sequences in all the evaluation metrics. We again noticed that the sensitivity was higher than specificity for all the DNN models, with differences ranging from 0.032 (VGG16 sensitivity: 0.858; specificity: 0.826) to 0.236 (ResNet-50 sensitivity: 0.899; specificity: 0.663).

**Table 3:** Performance of the deep neural network (DNN) models on individual magnetic resonance imaging (MRI) sequences: T1-weighted MRI (T1), T2-weighted MRI (T2), and gadolinium-contrast-enhanced T1-weighted MRI (T1c).

DNN model and magnetic resonance sequence		Sensitivity (95% CI)	Specificity (95% CI)	Accuracy (95% CI)	Area under the curve (95% CI)
**VGG^a^16**					
	T1	0.725 (0.696-0.753)	0.606 (0.562-0.648)	0.684 (0.660-0.708)	0.718 (0.689-0.747)
	T2	0.690 (0.660-0.719)	0.686 (0.644-0.727)	0.689 (0.665-0.713)	0.767 (0.740-0.794)
	T1c	0.874 (0.851-0.894)	0.540 (0.496-0.585)	0.759 (0.736-0.781)	0.770 (0.743-0.797)
**VGG19**					
	T1	0.804 (0.778-0.829)	0.448 (0.404-0.492)	0.681 (0.657-0.705)	0.692 (0.663-0.721)
	T2	0.743 (0.714-0.770)	0.554 (0.510-0.598)	0.678 (0.653-0.702)	0.741 (0.713-0.769)
	T1c	0.800 (0.773-0.825)	0.653 (0.610-0.694)	0.749 (0.726-0.771)	0.795 (0.769-0.821)
**ResNet^b^-50**					
	T1	0.782 (0.755-0.808)	0.584 (0.540-0.627)	0.714 (0.690-0.737)	0.732 (0.704-0.760)
	T2	0.833 (0.808-0.852)	0.525 (0.480-0.569)	0.727 (0.703-0.750)	0.762 (0.735-0.789)
	T1c	0.825 (0.799-0.848)	0.653 (0.610-0.694)	0.766 (0.743-0.787)	0.824 (0.800-0.848)
**Inception-v3**					
	T1	0.724 (0.695-0.752)	0.596 (0.552-0.639)	0.680 (0.656-0.704)	0.706 (0.677-0.735)
	T2	0.634 (0.603-0.665)	0.734 (0.693-0.772)	0.668 (0.644-0.693)	0.734 (0.706-0.762)
	T1c	0.769 (0.741-0.795)	0.732 (0.691-0.770)	0.756 (0.733-0.778)	0.831 (0.807-0.855)
**Inception-ResNet-v2**					
	T1	0.774 (0.746-0.800)	0.590 (0.546-0.633)	0.711 (0.687-0.734)	0.748 (0.720-0.776)
	T2	0.829 (0.804-0.852)	0.529 (0.484-0.573)	0.726 (0.702-0.748)	0.804 (0.779-0.829)
	T1c	0.812 (0.786-0.837)	0.722 (0.681-0.761)	0.781 (0.759-0.802)	0.841 (0.818-0.864)
**ERN-Net^c^**					
	T1	0.704 (0.674-0.732)	0.519 (0.474-0.563)	0.640 (0.615-0.665)	0.646 (0.615-0.676)
	T2	0.634 (0.603-0.665)	0.606 (0.562-0.648)	0.624 (0.599-0.649)	0.675 (0.645-0.705)
	T1c	0.803 (0.777-0.828)	0.643 (0.600-0.685)	0.748 (0.725-0.770)	0.807 (0.782-0.832)

^a^VGG: Visual Geometry Group.

^b^ResNet: residual neural network.

^c^ERN-Net: efficient radionecrosis neural network.

**Table 4:** Performance of different deep neural network (DNN) models on the T1^a^-T2^b^-T1c^c^-fused images for image-based classification.

DNN models	Sensitivity (95% CI)	Specificity (95% CI)	Accuracy (95% CI)	Area under the curve (95% CI)
VGG^d^16	0.858 (0.834-0.880)	0.826 (0.791-0.858)	0.847 (0.828-0.865)	0.864 (0.842-0.886)
VGG19	0.852 (0.828-0.874)	0.704 (0.662-0.744)	0.801 (0.780-0.821)	0.828 (0.804-0.852)
ResNet^e^-50	0.899 (0.879-0.918)	0.663 (0.620-0.704)	0.818 (0.797-0.837)	0.866 (0.844-0.888)
Inception-v3	0.844 (0.819-0.866)	0.716 (0.675-0.755)	0.800 (0.778-0.820)	0.845 (0.822-0.868)
Inception-ResNet-v2	0.925 (0.907-0.941)	0.755 (0.716-0.792)	0.867 (0.848-0.884)	0.913 (0.895-0.931)
ERN-Net^f^	0.820 (0.794-0.844)	0.789 (0.751-0.824)	0.809 (0.788-0.829)	0.915 (0.895-0.932)

^a^T1: T1-weighted magnetic resonance imaging (MRI).

^b^T2: T2-weighted MRI.

^c^T1c: gadolinium-contrast-enhanced T1-weighted MRI.

^d^VGG: Visual Geometry Group.

^e^ResNet: residual neural network.

^f^ERN-Net: efficient radionecrosis neural network.

### Subject-wise Classification

Table 5 shows the performance of different DNN models in the subject-wise classification task. Each of the 29 test subjects was considered as a single sample to be classified. In this experiment, the classification results of the images extracted from the same patient were averaged as the final output prediction of the subject. When the DNN models were evaluated on a per-subject basis by aggregating the subject’s image stack, the performance was further improved to an average sensitivity of 0.947 (SD 0.033), specificity of 0.817 (SD 0.075), accuracy of 0.903 (SD 0.026), and AUC of 0.938 (SD 0.022). Both ERN-Net and Inception-ResNet-v2 achieved the highest AUC of 0.958. While Inception-ResNet-v2 also had higher sensitivity and accuracy, ERN-Net had higher specificity. In particular, Inception-ResNet-v2 achieved a sensitivity of 100%, indicating that all recurrent tumors identified by Inception-ResNet-v2 were correct. VGG16 tied for the highest specificity (0.900) with ERN-Net and the highest accuracy (0.931) with Inception-ResNet-v2. The DNN models had higher sensitivity than specificity, except ERN-Net, implying that ERN-Net was less affected by the imbalanced distribution of necrosis and recurrent tumor samples on the subject level.

We also compared the performance of the DNN models to that of five neurosurgeons, with 7-26 years of experience, who were presented with the same multimodal MRI scans as used to test the DNN models. The neurosurgeons were not shown the pathological analysis reports and were requested to make diagnoses based on the MRI data alone. The neurosurgeons achieved an average sensitivity of 0.768 (SD 0.109), specificity of 0.360 (SD 0.089), and accuracy of 0.628 (SD 0.075), which were significantly worse than the DNN models when measured using *t* tests (*P*=.02 in sensitivity and *P*<.001 in specificity and accuracy).

**Figure 4:**
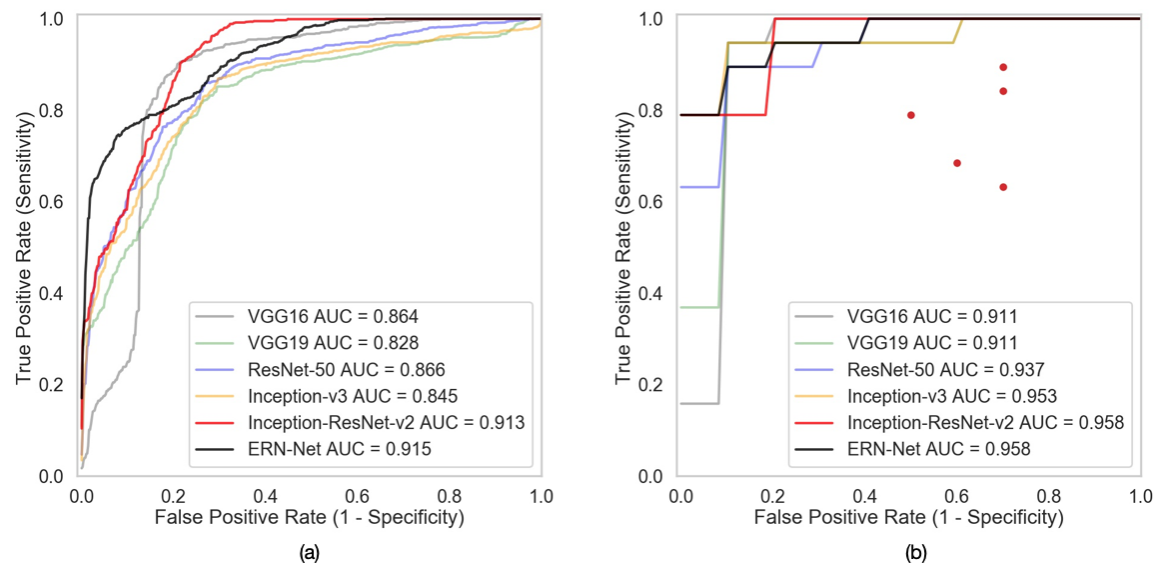
Plots showing (a) performance of the deep neural network (DNN) models on multimodal magnetic resonance imaging in the image-based classification task and (b) performance of the DNN models and neurosurgeons in the subject-based classification task. Performance of the DNN models was evaluated using the area under the curve (AUC) of the receiver operating characteristic curves, while the five neurosurgeons’ sensitivity and specificity scores are represented by the red dots. ERN-Net: efficient radionecrosis neural network; ResNet: residual neural network; VGG: Visual Geometry Group.

Figure 4 further shows the ROC curves and the AUC scores of the DNN models in the image-wise and subject-wise classification tasks. The red dots in Figure 4 (b) represent the neurosurgeons’ sensitivity and specificity scores.

**Table 5:** Performance of different deep neural network (DNN) models for subject-based classification; the T1^a^-T2^b^-T1c^c^-fused images were used as the input to the models.

DNN models	Sensitivity	Specificity	Accuracy	Area under the curve
VGG^d^16	0.947	0.9	0.931	0.911
VGG19	0.947	0.8	0.897	0.911
ResNet^e^-50	0.947	0.7	0.862	0.937
Inception-v3	0.947	0.8	0.897	0.953
Inception-ResNet-v2	1.000	0.8	0.931	0.958
ERN-Net^f^	0.895	0.9	0.897	0.958
All DNNs, mean (SD)	0.947 (0.033)	0.817 (0.075)	0.903 (0.026)	0.938 (0.022)
All neurosurgeons, mean (SD)	0.768 (0.109)	0.360 (0.089)	0.628 (0.750)	N/A^g^
*P* values for *t* tests between the DNNs and the neurosurgeons	.02	<.001	<.001	N/A

^a^T1: T1-weighted magnetic resonance imaging (MRI).

^b^T2: T2-weighted MRI.

^c^T1c: gadolinium-contrast-enhanced T1-weighted MRI.

^d^VGG: Visual Geometry Group.

^e^ResNet: residual neural network.

^f^ERN-Net: efficient radionecrosis neural network.

^g^N/A: not applicable. The diagnoses made by neurosurgeons are definite (ie, yes or no), unlike those made by the DNN models (eg, 30% yes or 70% no); therefore, the area under the curve cannot be computed without a probability distribution of predictions.

## Discussion

### Principal Findings

To the best of our knowledge, this is the first research on the application of DNN models to routine MRI scans for the purposes of automated differentiation between radiation necrosis and recurrent tumors. We found that T1c is the most informative routine MRI sequence for identifying radiation necrosis, which aligns well with many previous studies [1,2,5,6,13,15]. However, other routine MRI sequences, including T1 and T2, also provide useful and complementary information to T1c in characterizing the tumors and necrosis, as evidenced by the improved performance of the combined MRI sequences.

The proposed ERN-Net model achieved the highest AUC in both image-wise classification (0.915) and subject-wise classification (0.958), while being substantially smaller and faster compared to the other DNN models. Overall, the DNN models achieved better performance than the human experts. The most important advantage of the DNN models is that they have a higher discriminative power in recognizing radiation necrosis with a mean specificity of 0.817 (SD 0.075) compared to the mean specificity of 0.360 (SD 0.089) achieved by experienced neurosurgeons (*P*<.001).

**Figure 5:**
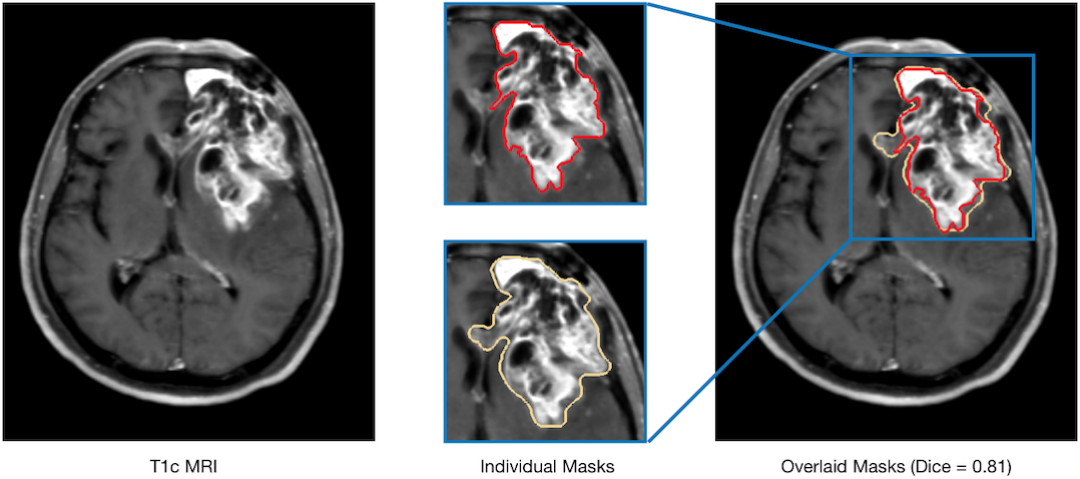
A T1c tumor image and its corresponding tumor masks created by two neuroradiologists independently, which shows the disagreement between annotators. MRI: magnetic resonance imaging; T1c: gadolinium-contrast-enhanced T1-weighted MRI.

Compared to previously reported machine learning methods, which were generally based on handcrafted features and user-defined classifiers [14-16], DNN models use an end-to-end approach to integrate feature learning and classification and, therefore, could eliminate the dependence on the selected feature descriptors and classifiers. Furthermore, the proposed method does not require manual drawing of the lesion, which is time-consuming and may result in interreader variance [17,39], as shown in Figure 5. We proposed a lesional slice identification approach to select the relevant slices instead of creating the lesion masks manually. This approach reduced the time required for annotating tumor masks and can also capture contextual spatial information of the perilesional tissues. Both the trained DNN models and the lesion slice identification module support cross-platform systems and can be seamlessly integrated into existing image analysis and reporting workstations within a hospital, aiming to generate differential diagnosis reports automatically. More importantly, the performance of the proposed method is substantially higher than that of the previously reported methods [14-16].

Currently, there exist other imaging techniques for differential diagnosis of recurrent tumor and radiation necrosis, such as MRS [7,8], PWI [9], DWI [10], and PET [11,12], yet none of them demonstrate sufficiently high efficacy for clinical use. A meta-analysis on PET showed that L-[*methyl*-^11^C]methionine (11C-MET) PET achieved promising results, with a pooled sensitivity and specificity of 0.880 (95% CI 0.850-0.910) and 0.850 (95% CI 0.800-0.890), respectively, and a summary receiver operating characteristic (SROC) score of 0.935 [12]. Another meta-analysis of 11C-MET PET showed an SROC score of 0.8914 [40]. Both PET meta-analysis studies showed a lower performance than the proposed method. In addition, the relative accessibility, radiation exposure, and higher cost of PET limit its clinical applicability. MRS demonstrated moderate diagnostic performance in differentiating glioma recurrence from radiation necrosis based on metabolite ratios, such as choline to creatinine and choline to N-acetylaspartate, and it is strongly recommended to combine MRS with other imaging technologies to improve diagnostic accuracy [3]. Previous studies on machine learning and imaging techniques have two notable limitations: first, the diagnoses included in many earlier studies were not pathologically confirmed; second, the sample sizes were too small. These limitations led to inconclusive findings, such that the differential diagnosis of tumor recurrence and necrosis is still a largely unsolved clinical problem [2,12]. To the best of our knowledge, the imaging data set (N=146) used in this study represents the largest cohort in the same kind of studies and includes pathologically confirmed diagnoses as ground truth labels; therefore, it is a more reliable data set to address this problem.

### Limitations and Future Work

There are also a few limitations of this study. Although we used a larger data set for the same analysis, it is still a relatively small data set compared to the generic image data sets used in the field of computer vision. This may potentially lead to overfitting or undertraining when training a DNN model. Furthermore, due to the retrospective nature of this study, the DNN models were only trained on an imbalanced data set with readily available 2D routine MRI sequences. The imbalanced distribution of samples may induce bias in the DNN model, leading to higher sensitivities but low specificities. Although we attempted to address this issue by weighting the samples during the training phase, the models still favor positive class over the negative class. It will be beneficial to extend the sample size by including data from other centers and using data augmentation methods to further improve and validate the proposed method. Other MRI sequences, such as FLAIR, PWI, DWI, and delayed-contrast MRI, and the 3D data set may potentially improve the classification performance of the DNN models. Last but not least, also due to the retrospective nature of this study, no glioma subtypes, such as astrocytoma, oligodendroglioma, and glioblastoma; molecular genetic features, such as isocitrate dehydrogenase and alpha thalassemia/mental retardation syndrome X-linked genes; nor 1p/19q chromosome co-deletion status [41,42] were included. These aspects should be investigated in future studies.

The proposed method has high clinical potential. Distinguishing glioma recurrence from radiation necrosis remains a critical challenge in clinical neuro-oncology. Misdiagnosing radiation necrosis as tumor recurrence may result in unnecessary surgery, whereas misdiagnosing tumor recurrence as radiation necrosis will delay the treatment of tumors. Currently, the differential diagnosis of radiation necrosis and recurrent tumor relies on histopathologic analysis, which requires biopsy or open surgery to gain tissue for the analysis. This study’s method proposes a sound alternative to the second surgery for the purpose of gaining tissue for histopathologic analysis, therefore avoiding invasive operations and lowering the risks to patients. In addition, up to now, there have been no clinical guidelines for preoperative diagnosis of glioma recurrence and radiation necrosis based on routine MRI sequences. Our study underlines important insights about the imaging of recurrent tumors and radiation necrosis through examining the radiological features learned by the DNN models; hence, it is likely to take an important role in formulating the guidelines for the differential diagnosis of recurrent lesions and for glioma follow-up.

### Conclusions

In this work, we demonstrated that DNN models based on multimodal MRI can differentiate radionecrosis from recurrent gliomas more effectively than models based on single MRI sequences; in addition, the DNN model’s performance is significantly better than that of the tested experienced clinicians on subject-wise diagnosis. Therefore, the proposed deep learning method, which does not depend on lesion segmentation or any handcrafted features, can be a useful tool for differentiating between radiation necrosis and recurrent tumors, with a high applicability potential in the clinical setting.

## Appendix

Multimedia Appendix 1 Description of the deep neural networks used in this study.

## Abbreviations

11C-MET: L-[*methyl*-^11^C]methionine
AUC: area under the curve
BTH-CMU: Beijing Tiantan Hospital, Capital Medical University
DNN: deep neural network
DWI: diffusion-weighted imaging
ERN-Net: efficient radionecrosis neural network
FLAIR: fluid-attenuated inversion recovery
FLIRT: Functional Magnetic Resonance Imaging of the Brain Linear Image Registration Tool
FMRIB: Functional Magnetic Resonance Imaging of the Brain
FSL: Functional Magnetic Resonance Imaging of the Brain Software Library
MRI: magnetic resonance imaging
MRS: magnetic resonance spectroscopy
NHMRC: National Health and Medical Research Council
PET: positron emission tomography
ResNet: residual neural network
ROC: receiver operating characteristic
ROI: region of interest
SROC: summary receiver operating characteristic
T1: T1-weighted magnetic resonance imaging
T1c: gadolinium-contrast-enhanced T1-weighted magnetic resonance imaging
T2: T2-weighted magnetic resonance imaging
VGG: Visual Geometry Group
