# Supplementary material for: Deep Learning Methodology for Differentiating Glioma Recurrence From Radiation Necrosis Using Multimodal Magnetic Resonance Imaging: Algorithm Development and Validation
Source: JMIR Med Inform. 2020 Nov 17;8(11):e19805. doi: 10.2196/19805 (PMC7708085; doi:10.2196/19805)
Supplement: Multimedia Appendix 1 [file medinform_v8i11e19805_app1.doc]

**Appendix: description of the deep neural networks used in this study.**

Deep neural network (DNN) models can be considered as mathematical functions with multi-million parameters. They can learn the feature representations from the training images and then to extract features from the test images for classification. All the DNN models used in this study are based on convolutional neural networks (CNN) that use a series of convolution functions to first combine nearby pixels into local features, and then aggregate these local features into global features for object detection or image classification [1].

VGG16 and VGG19 [2] are the winning DNN models in the ImageNet Large Scale Visual Recognition Challenge 2014 [3], which aimed to evaluate algorithms for object detection and image classification at large scale (more than 1 million images ranging over 1,000 categories). Simonyan and Zisserman [2] first demonstrate that the network depth has a significant impact on the model’s performance. Pushing the network depth to 16 (VGG16) and 19 (VGG19) can significantly improve the performance over prior-art configurations. A detailed description of the VGG16 and VGG19 models can be found in [2].

ResNet [4] is the winning algorithm on the ImageNet Challenge 2015. The ResNet architecture is designed to ease the difficulty of training DNNs by adding the skipping shortcut connections between one layer and a few stacked layers after that layer to fit a residual mapping, so that the network can avoid getting saturated rapidly and the depth of the network can be increased greatly, while maintaining low complexity. A few models based on the ResNet architecture (ResNet-34, ResNet-50, ResNet-101, ResNet-152) have been tested on the ImageNet dataset as well as many medical image datasets. A detailed description of the ResNet models can be found in [4].

Both Inception [5] and Inception-ResNet [6] models were developed by Google. The original Inception model achieved similar performance to VGG models on the ImageNet Challenge, but only used a fraction of parameters compared to VGG. The Inception model was later refined by incorporating batch normalization and factorization (Inception-v3) to enhance its training efficiency and generalizability to other image classification tasks. Inception-ResNet is an upgraded version of Inception-v3 that combines the Inception architecture with the residual connections, with significant improvement in training efficiency and better performance on the ImageNet data. A detailed description of the Inception-v3 and Inception-ResNet can be found in [5,6].

The proposed Efficient Radionecrosis Neural Network (ERN-Net) consists of 9 convolutional modules, with a convolutional layer, batch normalization and ReLU unit in each module. The inverted linear bottleneck layers [7] are inserted in the middle 7 convolutional modules to enhance the low-dimensional compressed representation, hence reducing the number of operations and memory needed by the model while retaining the same accuracy. This architecture, equivalent to the EfficientNet-B1 which has been thoroughly discussed by Tan and Le [8], was also tested on the ImageNet dataset and outperformed Inception-v3 and ResNet-152 while being 7.6 times and 3 times smaller than these two models, respectively. A detailed description of the EfficientNet-B1 can be found in [8], and the TensorFlow implementation of it is available online [[1]](#footnote-2).

In this study, all these models were implemented using the TensorFlow framework [9] and pre-trained using the ImageNet dataset [3] which consists of 1.28 million RGB images across 1,000 classes. The pre-trained weights were imported from the Keras library [10]. We replaced the default output Dense layer which has 1,000 neurons with a new Dense layer for all the models. A dropout layer [11] was added on the output layer before the output layer to control overfitting. All layers in the networks were set to 'trainable' in our implementation. To address the imbalanced sample distribution, we assigned different weights to the classes during the training phase based on the ratio of the number of samples in each class to the total number of samples scaled by the number of classes (Necrosis: 1.5; Recurrence: 0.75).

For the VGG16 and VGG19 models, the stochastic gradient descent (SGD) optimizer with back propagation [12] was used with a batch size of 16, learning rate of 1e-5, decay rate of 1e-6, momentum of 0.9, validation split ratio of 0.2, and 50 epochs. For the other models, the Adam optimizer [13] was used with the same batch size, learning rate, decay rate, validation split and number of epochs.

**References**

1. Y. LeCun, Y. Bengio, and G. Hinton, “Deep learning,” *Nature*, vol. 521, no. 7553, pp. 436–444, 2015.
2. Simonyan K, Zisserman A. Very Deep Convolutional Networks for Large-Scale Image Recognition. International Conference on Learning Representations (2015).
3. Russakovsky O, Deng J, Su H, Krause J, et al. ImageNet Large Scale Visual Recognition Challenge. Int. Journal of Computer Vision 2015; 115(3): 211-252.
4. He K, Zhang X, Ren S, Sun J. Deep residual learning for image recognition. International Conference on Computer Vision and Pattern Recognition (2016).
5. Szegedy C, Vanhoucke V, Ioffe S, Shlens J. Rethinking of Inception Architecture for Computer Vision. CVPR (2016).
6. Szegedy C, Ioffe S, Vanhoucke V. Inception-v4, Inception-ResNet and the Impact of Residual Connections on Learning. In: arXiv:1602.07261 (2016).
7. M. Sandler, A. Howard, M. Zhu, A. Zhmoginov, and L. Chen, MobileNetV2: Inverted Residuals and Linear Bottlenecks. CVPR 2018, pp. 4510–4520.
8. M. Tan and Q. V Le, EfficientNet: Rethinking Model Scaling for Convolutional Neural Networks, CVPR 2019, pp. 6105–6114.
9. Abadi M, Barham P, Chen J, Chen Z, et al. TensorFlow: A System for Large-Scale Machine Learning. USENIX Symposium on Operating Systems Design and Implementation (2016).
10. Chollet F, et al. Keras. [https://keras.io](https://keras.io/). (2015).
11. Srivastava N, Hinton G, Krizhevsky A, Sutskever I, Salakhutdinov R. Dropout: A Simple Way to Prevent Neural Networks from Overfitting. Journal of Machine Learning Research 2014; 15: 1929-1958.
12. LeCun Y, Boser B, Denker JS, Henderson D, et al. Backpropagation Applied to Handwritten Zip Code Recognition. Neural Computation 1989; 1: 541-551.
13. Kingma DP, Ba J. Adam: A Method for Stochastic Optimization. International Conference for Learning Representations (2015).

1. https://github.com/qubvel/efficientnet [↑](#footnote-ref-2)
